# CD271-selected mesenchymal stem cells from adipose tissue enhance cartilage repair and are less angiogenic than plastic adherent mesenchymal stem cells

**DOI:** 10.1038/s41598-019-39715-z

**Published:** 2019-02-28

**Authors:** Nupur Kohli, Ibtesam R. T. Al-Delfi, Martyn Snow, Takumi Sakamoto, Tsuyoshi Miyazaki, Hideaki Nakajima, Kenzo Uchida, William E. B. Johnson

**Affiliations:** 1 Life and Health Sciences, Aston University, Birmingham, B4 7ET UK; 2 Regenerative Biomaterials Group, RAFT Institute, Leopold Muller Building, Mount Vernon Hospital, Northwood, London HA6 2RN UK; 3 Al-Toosi Private University College, Nursing Department, Al-Saad Quarter, Al-Muthanaa Street, Al-Najaf, Iraq; 4 Royal Orthopaedic Hospital NHS Foundation Trust, Birmingham, B31 2AP UK; 5 Department of Orthopaedics and Rehabilitation Medicine, Faculty of Medical Sciences University of Fukui, Matsuoka Shimoaizuki, Eiheiji, Fukui Japan; 6 Faculty of Medicine, Dentistry and Life Sciences, University of Chester, Chester, CH1 4BJ UK

## Abstract

CD271 is a marker of bone marrow MSCs with enhanced differentiation capacity for bone or cartilage repair. However, the nature of CD271+ MSCs from adipose tissue (AT) is less well understood. Here, we investigated the differentiation, wound healing and angiogenic capacity of plastic adherent MSCs (PA MSCs) versus CD271+ MSCs from AT. There was no difference in the extent to which PA MSCs and CD271+ MSCs formed osteoblasts, adipocytes or chondrocytes *in vitro*. In contrast, CD271+ MSCs transplanted into athymic rats significantly enhanced osteochondral wound healing with reduced vascularisation in the repair tissue compared to PA MSCs and control animals; there was little histological evidence of mature articular cartilage formation in all animals. Conditioned medium from CD271+ MSC cultures was less angiogenic than PA MSC conditioned medium, and had little effect on endothelial cell migration or endothelial tubule formation *in vitro*. The low angiogenic activity of CD271+ MSCs and improved early stage tissue repair of osteochondral lesions when transplanted, along with a comparable differentiation capacity along mesenchymal lineages when induced, suggests that these selected cells are a better candidate than PA MSCs for the repair of cartilaginous tissue.

## Introduction

Regenerative medicines using stem cells and/or tissue engineering approaches are expected to play a major role in the development of new healthcare in the 21^st^ century, with much current interest and research activity. However, a major problem to this approach, especially if using autologous stem cells sourced from adult tissues remains in optimising which cells are best applied for different pathological conditions and tissues. An example of this can be found in the development of treatment strategies for osteochondral defects, which is problematic as the damage extends to the subchondral bone and the repair responses demands the healing and reformation of two connective tissue types, i.e. cartilage and bone^1,2^. Current surgical treatment include the use of osteochondral auto/allografts, which often lead to poor subchondral bone regeneration with superficial fibrocartilage formation^3–5^. Repairing osteochondral damage by transplanting mesenchymal stromal/stem cells (MSCs), which have the capacity to differentiate into chondrocytes and osteoblasts forming new cartilage and bone, respectively, has shown some promise^6–8^. However, MSCs are often isolated from bone marrow, which can mean a painful operative procedure and morbidity^9^, and recent data suggests that rather than differentiating to form chondrocytes or osteoblasts, transplanted MSCs may repair tissues by exerting paracrine effects on endogenous cells to increase wound healing processes^10,11^, e.g. by stimulating angiogenic responses in bone, but not cartilage^12,13^. Hence, the use of MSCs for the repair of deep cartilaginous wounds highlights how there is a need to optimise adult stem cell-based therapies as well as identify potential mechanisms of action.

For this reason, much current interest in MSC-based regenerative medicines has been examining defined subpopulations of MSCs from the heterogeneous pools of cells present in bone marrow and different tissue sources^14–17^. The cell surface markers CD73 and CD105 were reported to select bone marrow MSCs (BM MSCs) that have increased chondrogenic differentiation capacity compared to non-selected BM MSCs, which are routinely isolated based on their capacity to adhere to tissue culture plastic^16,18^. In addition, CD271 was proposed as a marker of BM MSCs with multi-potential differentiation capacity, but with increased capacity to form bone^14,19–22^. Alternative tissue sources of MSCs have also been investigated. Adipose tissue, in particular, is an attractive autologous source as it is often abundant in human adults and relatively easy to harvest compared to bone marrow^23–25^. However, whether adipose tissue-derived MSCs (AT MSCs) can form cartilage and bone is unclear, with research demonstrating reproducible differentiation along chondrocytic and osteogenic lineages *in vitro*, but with more uncertain results seen *in vivo*^26–29^. In addition, the properties of selected AT MSC subpopulations have been less extensively researched than BM MSCs, although two studies have reported that CD271-selected AT MSCs have an enhanced proliferative capacity compared with unsorted plastic adherent (PA MSCs), which is how MSCs are generally isolated from tissue harvests, with differentiation capacity to form chondrocyte-like cells, osteoblasts and adipocytes *in vitro*^30,31^. Importantly, whether CD271-selected AT MSCs have enhanced capacity to heal cartilage or bone defects, or differential paracrine wound healing activity, is currently unknown. Therefore, the aim of this study was to investigate the wound healing potential of AT MSCs selected on the basic of plastic adherence (PA MSCs) versus immunopositivity for CD271 (CD271+ MSCs) in a rodent model of osteochondral tissue damage, using a commercially available cell scaffold for cartilage repair as a delivery system^8^. In addition, we have examined the differentiation capacity and angiogenic activity of PA MSCs versus CD271+ MSCs *in vitro*.

## Results

### CD profiling and *in vitro* differentiation of PA MSCs and CD271+ MSCs

**Figure 1:**
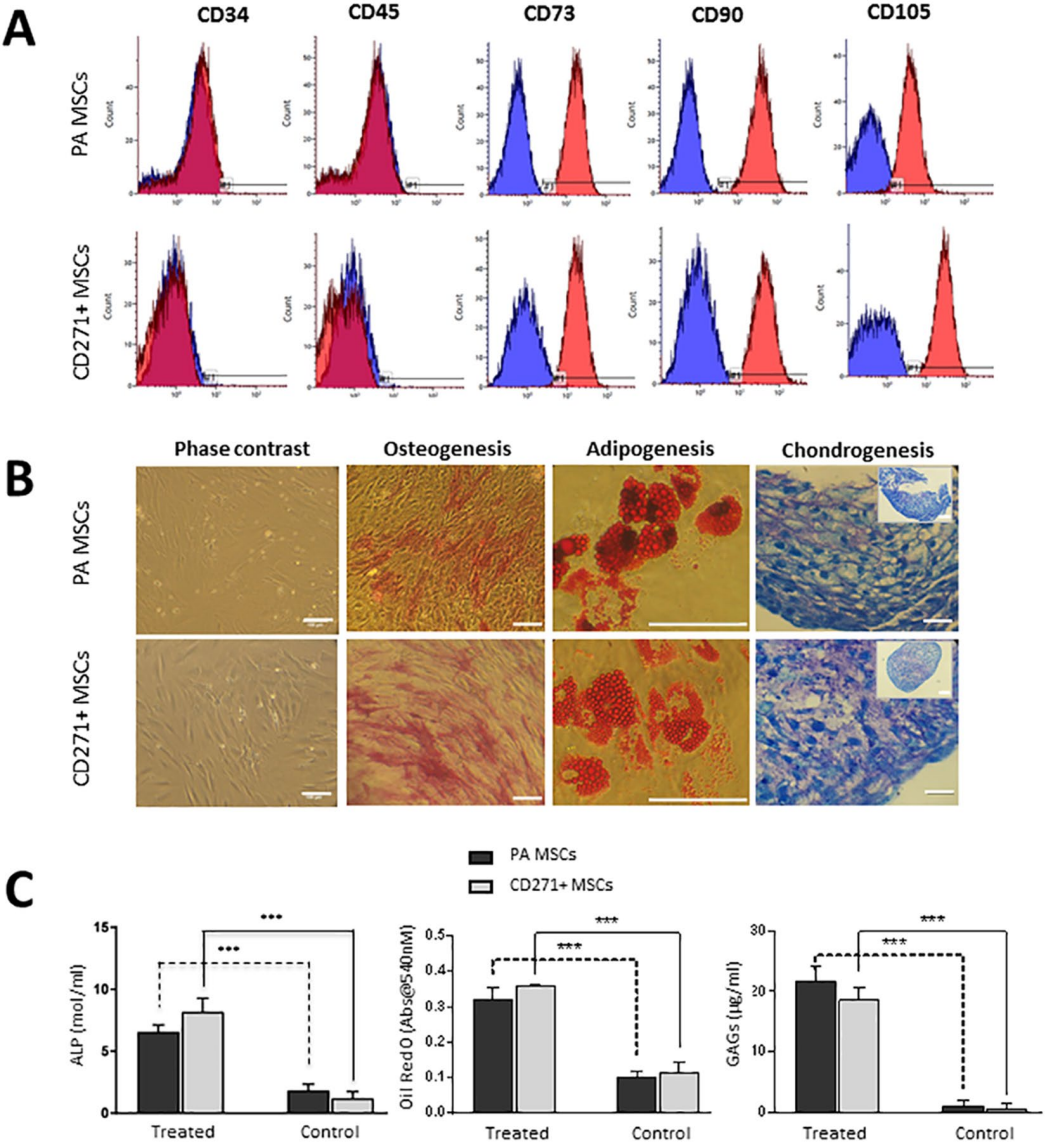
Differentiation and CD profiles of PA MSCs and CD271+ MSCs. (**A**) Both cell types showed immunopositivity for CD73, CD90 and CD105 and immunonegativity for CD34 and CD45. (**B**) PA MSCs and CD271+ MSCs show similar qualitative differentiation potential as represented by positive alkaline phosphatase staining (pink-red cells) for osteogenesis, positive Oil Red O staining of lipid droplets for adipogenesis (orange-red droplets) and metachromatic toluidine blue staining of glycosaminoglycans (GAGs) for chondrogenesis. Scale bar = 100 µm. (**C**) Increased levels of alkaline phosphatase, Oil Red O and GAGs were secreted by both PA MSCs and CD271+ MSCs when induced to differentiate down the osteogenic, adipogenic and chondrogenic lineages, respectively, compared to the undifferentiated controls. Data shown are means ± SEMs of n = 3 for Oil Red O and GAGs levels; means ± SDs for ALP levels.

Culture-expanded PA MSCs and CD271+ MSCs showed immunopositivity for MSC specific cell-surface antigens, i.e., CD73, CD90 and CD105, to a similar extent, and were immunonegative for haematopoietic markers, i.e., CD34 and CD45 (Fig. 1A). These adherent cells were of stromal appearance and differentiated down the three mesodermal lineages, as indicated by positive alkaline phosphatase staining for osteogenesis, positive Oil Red O staining of lipid vacuoles for adipogenesis and metachromatic toluidine blue staining of paraffin-sections of cell pellets for chondrogenesis (Fig. 1B). Significant increases (p < 0.001) were seen in the levels of alkaline phosphatase (ALP), Oil Red O accumulation and glycosaminoglycan (GAGs) secreted in differentiated cell cultures compared to undifferentiated cultures (Fig. 1C). There were no significant differences in the extent of PA MSC and CD271+ MSC differentiation, as delineated by these measures, and no obvious differences in PA MSC and CD271+ MSC numbers, as depicted through cell confluence or pellet size (and H&E staining of pellet sections); however, it will be important to normalise these differentiation outcome measures to confirmed cell numbers in future studies.

### The effects of PA MSCs and CD271+ MSCs seeded Alpha Chondro Shield on cartilage repair: gross morphology at 3 weeks post-transplantation

**Figure 2:**
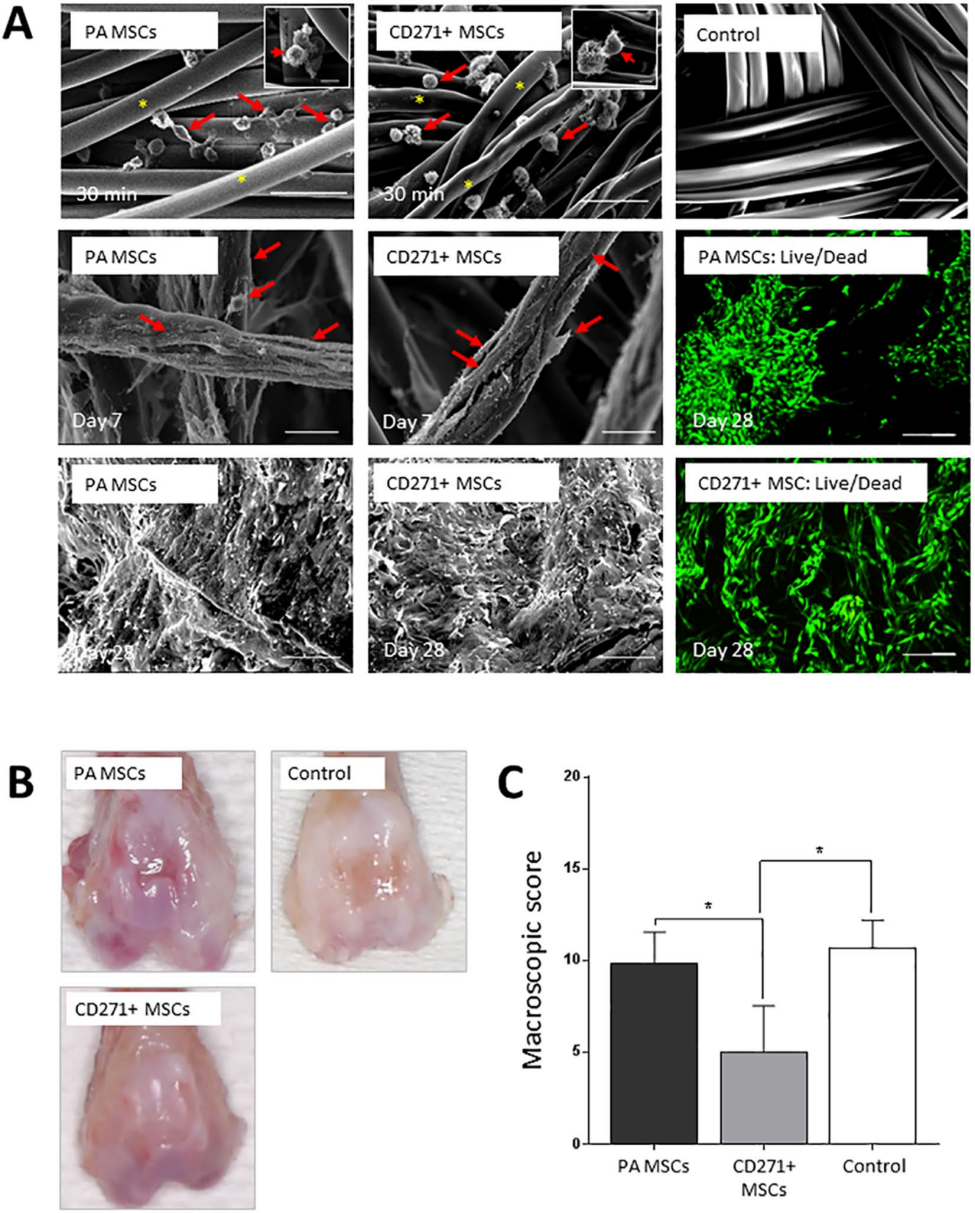
Gross morphology and the wound repair of defects. (**A**) Representative SEM images of cell-seeded Alpha Chondro Shield are shown. Both PA MSCs and CD271+ MSCs (red arrows) were attached to the fibres (yellow asterisks) of polyglycolic acid at 30 minutes post-seeding. The Alpha Chondro Shield scaffold alone control is also shown. With further time in culture (days 7 and 28), the PA MSCs and CD271+ MSCs became more fibroblastic in appearance and filled the scaffold. LIVE/DEAD staining was performed on long-term cultures, where all cells appeared to be viable (green fluorescence). Scale bars = 50 µm for top and bottom panel SEM; scale bars = 25 µm for mid panel SEM and LIVE/DEAD images. Insets (top panels) show high magnification images of cells firmly attached to the scaffold fibres. Inset scale bar = 10 µm. (**B**) Representative images are shown of the gross morphology of the defects transplanted with PA MSCs, CD271+ MSCs and Alpha Chondro Shield alone. Gross examination revealed a glossy white and well-integrated repair tissue in the animals that received CD271+ MSCs, but not in animals that received PA MSCs or Alpha Chondro Shield alone (control). (**C**) The overall macroscopic scores of defects transplanted with CD271+ MSCs were significantly better than the defects transplanted with PA MSCs (p value = 0.043) or control (p value = 0.039). Data shown are means ± SDs.

SEM demonstrated that PA MSCs and CD271+ MSCs incorporated within a cell scaffold consisting of fibres of polyglycolic acid (PGA), called Alpha Chondro Shield, within 30 minutes of seeding. There was no difference between the prevalence of PA MSCs and CD271+ MSCs within the scaffold and both cell types showed firm attachment to the PGA fibres, with a few cells showing a flattened morphology, although most remained rounded at this stage (Fig. 2A, upper panels). Therefore, this time point (30 minutes) was used to ensure cell adhesion and incorporation prior to implantation of the cell-seeded scaffolds into osteochondral defects that had been created simultaneously in athymic rats. Scaffolds were implanted alone also, i.e., in culture medium but without prior cell seeding, as a control. To confirm the biocompatibility of the Alpha Chondro Shield scaffold for MSC adhesion and growth, we performed SEM and LIVE/DEAD staining of the cell-seeded scaffolds after they had been maintained for 7 days and 28 days *in vitro* in culture medium. As shown (Fig. 2A, lower panels), both the PA MSCs and the CD271+ MSCs became fibroblastic, remained adherent to the PGA fibres and proliferated to completely cover and fill the Alpha Chondro Shield scaffold; furthermore, there was no evidence of any cell death.

Gross examination of the osteochondral defects at 3 weeks post-transplantation revealed a well-integrated glossy white repair tissue in the defects transplanted with CD271+ MSCs, which appeared less vascularised, better integrated and with a greater defect fill (Fig. 2B, Table 1). In the defects transplanted with PA MSCs, a depressed repair tissue with distinct defect edges was observed. Moreover, the border area of the defect was clearly distinguishable and depressions were obvious in those lesions of the scaffold alone control group (Fig. 2B, Table 1). The animals transplanted with CD271+ MSCs had a significantly better overall macroscopic repair score (*p* < 0.05) compared to the animals transplanted with PA MSCs or with the Alpha Chondro Shield scaffold alone. Animals transplanted with CD271+ MSCs scored 5.0 ± 2.5, whereas those transplanted with PA MSCs scored at 9.8 ± 1.7, and the control group was scored at 10.6 ± 1.5 (Fig. 2C) (means ± SD).

**Table 1:** Macroscopic scores for individual parameters of cartilage repair.

Macroscopic parameter	PA MSCs	CD271+ MSCs	Control
Colour of the repair tissue	2.3 ± 0.5	1.3 ± 0.8	2.3 ± 0.6
Blood vessels in the repair tissue	1.7 ± 0.8	**0.8** **±** 0.8*	**2.7** **±** **0.6**
Surface of the repair tissue	**2.2** **±** **0.4**	**1.2** **±** 0.4**	2.0 ± 0.0
Defect fill	**1.7** **±** **0.5**	**0.5** **±** 0.5*	1.3 ± 0.6
Degeneration of adjacent cartilage	2.0 ± 0.6	1.3 ± 0.5	2.3 ± 0.6

Bold text indicates statistical significant differences with asterisks displayed on the lower scores. Data shown are means ± SDs. * < 0.05; ** < 0.01.

### The effects of PA MSCs and CD271+ MSCs seeded Alpha Chondro Shield on cartilage repair: histological outcomes at 3 weeks post-transplantation

**Figure 3:**
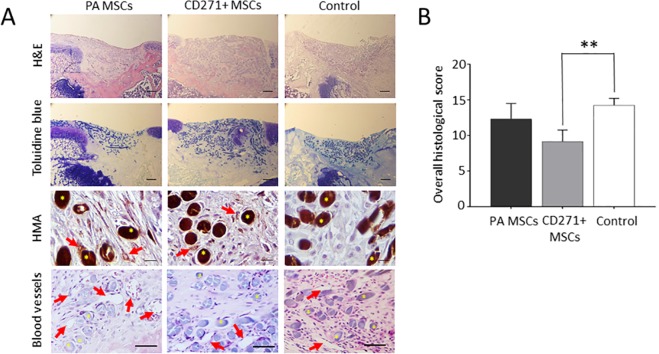
Histological assessment of the cartilaginous repair tissue. (**A**) Representative images are shown of the tissue sections of rat knees stained with haematoxylin and eosin (H&E), toluidine blue and following immunolocalisation for human mitochondrial antigen (HMA). A greater extent of defect fill with areas of prominent metachromatic staining of GAG deposition was observed in the defects transplanted with CD271+ MSCs compared to defects transplanted with PA MSCs and the Alpha Chondro Shield scaffold alone. The presence of human cells was confirmed by immunolocalization of HMA as indicated by brown punctate staining (red arrows) around the PGA fibres (yellow asterisks) of the scaffold in the cell-treated groups, but not in scaffold alone group. Greater vascularisation was seen in the defects transplanted with PA MSCs and the Alpha Chondro Shield scaffold alone compared to defects transplanted with CD271+ MSCs. Scare bars: H&E top row and toluidine blue = 200 µm; HMA = 10 µm; H&E bottom row = 50 µm. (**B**) There was a significantly lower overall histological score, indicative of better repair tissue, in defects transplanted with CD271+ MSCs compared with the control group. Data shown are means ± SDs. **p < 0.01.

Defect fill, observed by H&E and toluidine blue staining, was greater in those defects that were transplanted with CD271+ MSCs compared to defects transplanted with PA MSCs (Fig. 3A, upper panels). A firm attachment of the scaffold to the underlying subchondral region or trabecular bone was seen in all cases. Defects transplanted with scaffold alone were infiltrated with a cell-rich fibrous tissue. The repair tissue within the defects that were transplanted with MSCs showed some localised toluidine blue metachromasia, most notably for those animals transplanted with CD271+ MSCs, whereas the cell-rich fibrous tissue in the control animals transplanted with Alpha Chondro Shield scaffolds alone showed no metachromasia (Fig. 3A, upper-mid panels). Immunolocalisation for human mitochondrial antigen (HMA) was observed to a similar extent in the repair tissues of the PA MSCs and CD271+ MSC transplanted animals, but was absent in the Alpha Chondro Shield alone control group (Fig. 3A, lower-mid panels). HMA-positive cells were detected embedded in the fibrous ECM surrounding the scaffold fibres. Due to the nature of mitochondrial staining, it was not possible to quantify the exact number of HMA-immunopositive cells. Animals transplanted with PA MSCs or the Alpha Chondro Shield alone showed a greater vascularisation within the repair tissue compared to the animals transplanted with CD271+ MSCs (Fig. 3A, lower panels). Scoring for histological criteria demonstrated that in those animals transplanted with CD271+ MSCs there was a markedly and significantly greater thickness of defect fill with cartilaginous/fibrous tissue compared with those animals transplanted with the PA MSCs or the control group (*p* < 0.05) and significantly fewer blood vessels in the CD271+ MSC-transplanted group compared with those animals transplanted with the PA MSCs (*p* < 0.05), with little difference between groups seen in all other criteria (Table 2). Furthermore, there was a significant difference seen between the overall histological score for the CD271+ MSC transplanted group compared with the control group (*p* < 0.01), which was not seen for the PA MSC transplanted group versus the control group (Fig. 3B). The overall histological scores for CD271+ MSCs transplanted animals was 9.2 ± 1.6; for PA MSC-transplanted animals was 12.3 ± 2.1, and for the Alpha Chondro Shield alone control group was 14.3 ± 1.0 (means ± SD).

**Table 2:** Histological scores of individual parameters for cartilage repair.

Histological parameter	PA MSCs	CD271+ MSCs	Control
Cell morphology	2.0 ± 0.0	2.0 ± 0.0	3.0 ± 0.0
Matrix staining	1.5 ± 0.5	1.7 ± 0.5	2.0 ± 0.0
Surface regularity	1.3 ± 0.5	1.5 ± 0.5	1.7 ± 0.5
Thickness	**1.0** **±** **0.6**	**0.0** **±** 0.0*	**1.3** **±** **0.5**
Integration	0.7 ± 0.5	0.3 ± 0.5	0.5 ± 0.6
Blood vessels	**3.7** **±** **0.5**	**1.2** **±** 0.7*	3.2 ± 0.5
Foreign body giant cells	2.2 ± 0.7	2.3 ± 1.2	2.8 ± 0.5

The histological scores for all criteria in the different experimental groups, where 0 = “best repair” and 4 = “worst repair”. The scores for defect fill (thickness) was significantly different in the CD271+ MSC-transplanted group compared to the PA MSC-transplanted group and the control group. Furthermore, there were significantly fewer blood vessels present in the repair tissue of the CD271+ MSC-transplanted animals compared with the PA MSC-transplanted animals. Bold text indicates statistical significant differences, with the asterisks displayed on the lower scores. Data shown are means ± SDs. * < 0.05

### *In vitro* paracrine angiogenic activity of PA MSCs and CD271+ MSCs

**Figure 4:**
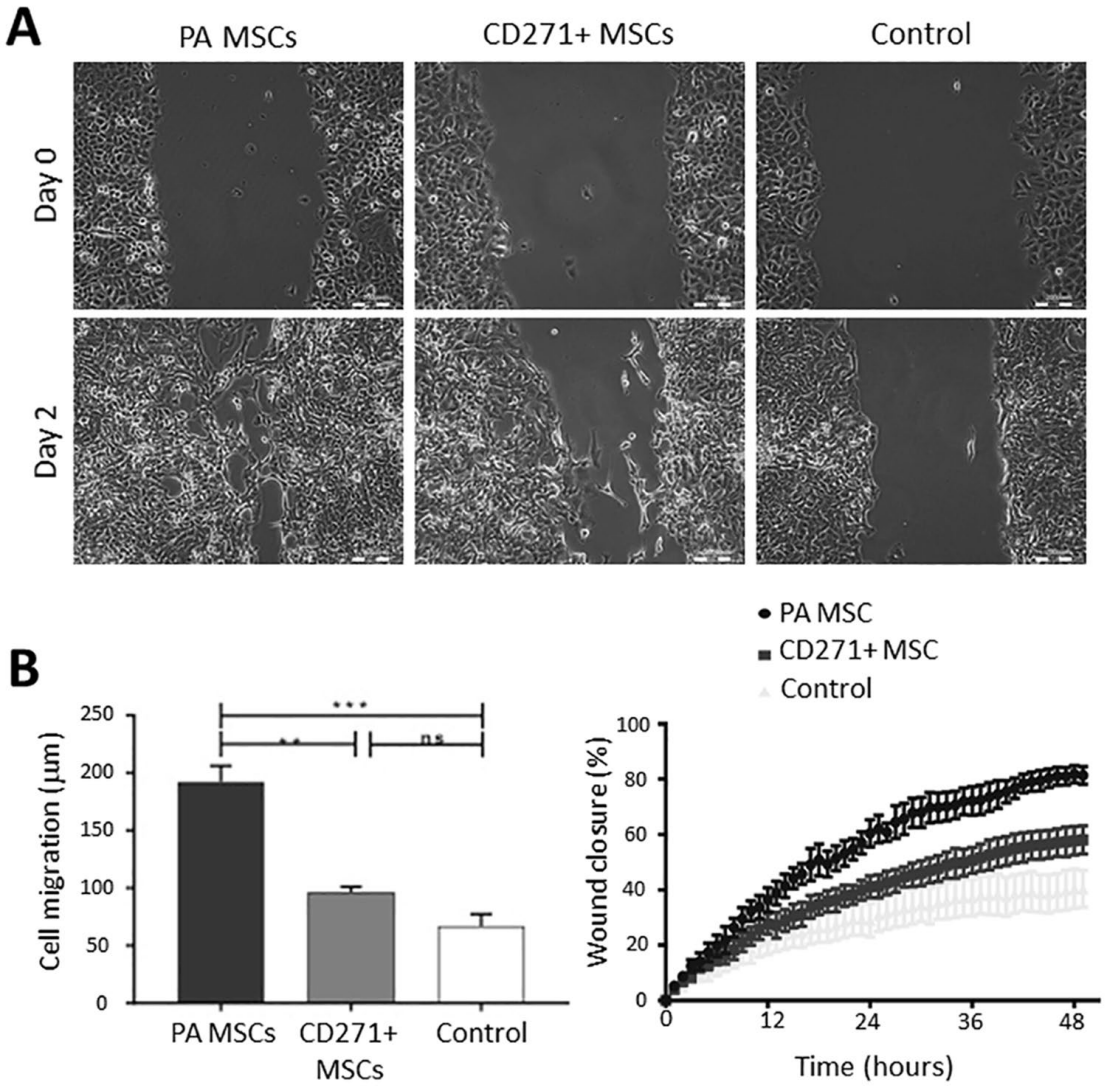
The effects of conditioned medium from PA MSCs and CD271+ MSCs on EA.hy926 endothelial cell migration. (**A**) Live cell imaging was used to determine the rate of endothelial cell migration in scratch assays in PA MSC conditioned medium versus CD271+ MSC conditioned medium and non-conditioned control medium. As shown, there was a significant increase in endothelial migration in PA MSC conditioned medium versus both other groups, with CD271+ MSC conditioned medium proving no greater than control conditions. (**B**) This difference in endothelial cell migration was further evidenced by the rate of wound closure in these scratch assays, where the rate of closure in CD271+ MSC conditioned medium was significantly slower compared with the PA MSC conditioned medium (*p* < 0.01). Data shown are means ± SEMs.

**Figure 5:**
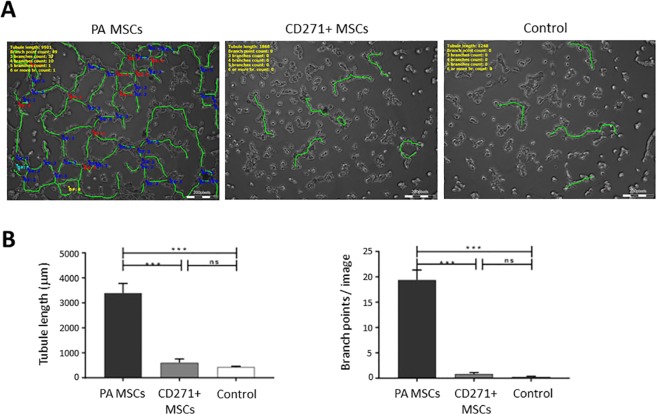
The effects of conditioned medium from PA MSCs and CD271+ MSCs on EA.hy926 endothelial cell tubule formation. (**A**) There was greater formation of tubule-like structures when EA.hy926 cells were treated with PA MSC conditioned medium compared to CD271+ MSC conditioned medium and non-conditioned control medium. (**B**) There was a significant difference (*p* < 0.001) in tubule length and branch points/image formed by EA.hy926 endothelial cells when treated with PA MSC conditioned medium compared to CD271+ MSC conditioned medium and non-conditioned control medium. Data shown are means ± SEMs.

The stimulatory activity of conditioned medium from MSC cultures versus control (non-conditioned) medium on EA.hy926 endothelial cell migration and proliferation was determined using phase contrast microscopy and image analysis over time of scratch wound assays (Fig. 4A). The extent of wound closure was significantly reduced in conditioned medium from CD271+ MSC cultures compared to the effects of conditioned medium from PA MSC cultures (*p* < 0.01). In these scratch wound assays, the extent to which the original wound width had closed at 48 hours after scratching was 81.4 ± 3.2% in PA MSC conditioned medium, 58 ± 5.1% in CD271+ MSC conditioned medium, whilst only 40.3 ± 6.7% in control medium (Fig. 4B). There was no significant difference in the original scratch wound widths at time 0 (data not shown). Matrigel assays of EA.hy926 endothelial tubule formation in MSC culture-conditioned medium versus control medium corroborated the angiogenic effects of the scratch assays, where significant increases (*p* < 0.001) in endothelial tubule length and the number of endothelial tubule branch points (also indicative of new blood vessel formation) were seen when EA.hy926 endothelial cells were maintained in PA MSC conditioned medium compared to either CD271+ MSC conditioned medium or control medium (Fig. 5A,B).

## Discussion

CD271 selected MSCs from various sources including BM^32^, AT^30,33^ and synovium^18^ have shown potential for their use in regenerative medicine. *In vitro* studies suggest that isolating cells that are immune-positive for CD271 results in a homogenous population that also co-expresses CD73, CD90 and CD105^18,32,34^. CD271+ MSCs from BM have previously been shown to give rise to superior cartilage formation *in vivo* compared to PA MSCs^35^. Here, osteochondral  defects transplanted with AT derived CD271+ MSCs showed macroscopically superior repair tissue compared to defects transplanted with PA MSCs and the control group of scaffold alone. Histologically, all defects showed fibrous cartilaginous repair tissue, but localised metachromatic toluidine blue staining was seen more prominently in the repair tissue of defects treated with CD271+ MSCs. In addition, there was a significantly greater fill of cartilaginous/fibrous tissue formed in the defect area after CD271+ MSC transplantation, which was markedly less vascularised than that formed after PA MSC transplantation or in control (scaffold alone) animals. Furthermore, only CD271+ MSC-transplanted animals had an overall histological score that was significantly different to that of control animals. These data suggest that CD271+ MSCs from adipose tissue, similar to CD271+ MSCs from bone marrow, are better candidate cells for the promotion of cartilage repair.

For complete healing of full-thickness osteochondral defects, it is essential that the trabecular and subchondral bones also are fully restored in addition to resurfacing of the lesion with articular cartilage. Therefore, it is advantageous for transplanted candidate cell type(s) to have the ability to differentiate to form bone- and cartilage-forming cells. Our analysis of MSC differentiation *in vitro* suggested that PA MSCs and CD271+ MSCs from AT had a similar capacity to become osteoblasts or chondrocytes. However, although there were no significant differences in the extent of PA and CD271+ MSC differentiation, as delineated by alkaline phosphatase activity, Oil Red O accumulation, or the release of GAGs, it will be important to normalise these differentiation outcome measures to confirmed cell numbers in future. More importantly, in both cell transplantation animal groups there was little evidence of mature hyaline cartilage or new bone formation. In addition, after staining for the human mitochondrial antigen (HMA) in the excised osteochondral tissues, to identify the presence of transplanted human MSCs in the rats, there was no evidence of HMA immunopositive cells being encased in hyaline-like cartilage or in bone, where instead HMA-positive cells were seen in fibrous tissues and around the PGA scaffold material. This suggests that neither the transplanted PA MSCs nor the transplanted CD271+ MSCs differentiated to form mature chondrocytes or osteoblasts.

The lack of evident transplanted MSC differentiation *in vivo* could be due to an early time point of analysis, i.e., at 3 weeks post-transplantation and further research is required to examine the long-term fate of these cells. Wakitani *et al*., have previously listed the sequence of events that are likely to take place following transplantation of cell-scaffolds into a full thickness defect^36^. In rats, such osteochondral lesions heal without intervention within 6–8 weeks^28,37–40^. Wakitani *et al*. hypothesized that vascular infiltration into the defect causes host-derived MSCs to undergo osteogenesis and restore the osseous tissue. This happens only up to the natural junction between the host cartilage and subchondral bone, as the distal part of the defect is under the influence of factors present in the synovial cavity where the osteochondral progenitor cells give rise to the cartilage^36^. The fact that there was little evidence of mature hyaline-like cartilage in either of the MSC transplanted groups also may be due to our use of subcutaneous AT as a cell source. Mochizuki and co-workers reported that subcutaneous AT is not a suitable source of MSCs for cartilage repair as subcutaneous AT MSCs have lower chondrogenic potential compared to synovium-derived MSCs^39^. In addition, in a donor-matched comparison, infrapatellar fat pad MSCs were reported to have a higher chondrogenic potential than subcutaneous AT MSCs^40^.

The differences in the effects of PA MSCs and CD271+ MSCs on cartilage repair may be associated with differences in angiogenesis. AT MSCs have previously been shown to secrete angiogenic factors that are inhibitory to cartilage-like ECM deposition^28^. In the current study, vascular invasion was seen in the repair tissue of both of the MSC-treated groups as well as the scaffold-alone transplanted group, which may have prevented hyaline-like ECM deposition. However, the CD271+ MSC transplanted group contained a comparatively and significantly lower number of blood vessels compared to PA MSCs and the control group. Other studies also have previously shown that articular cartilage GAGs inhibit endothelial cell adhesion and migration^41–43^. It may be that CD271+ MSCs induced an early cartilage-like repair response comprising of GAGs that inhibit blood vessel formation, whereas PA MSCs did not induce the deposition of GAGs.

To explore further the differences seen in the blood vessel invasion of the repair tissue formed in MSC-transplanted osteochondral lesions, we examined the comparative effects of conditioned medium from PA MSCs and CD271+ MSCs versus non-conditioned control medium on EA.hy926 endothelial cells *in vitro*. Our data demonstrates that PA MSCs secreted factors into culture that were angiogenic, promoting endothelial cell migration and tubule formation, whilst conditioned medium from CD271+ MSC cultures did not. These findings are consistent with our *in vivo* data and support the conclusion that CD271+ MSCs are less angiogenic than PA MSCs. Furthermore, the data supports the suggestion that implanted CD271+ MSCs may have enhanced cartilaginous wound repair through MSC-mediated ECM deposition, albeit mostly fibrous in nature, whilst having little angiogenic effect on endothelial cells. Previous studies have reported that MSCs can act as therapeutic agents through their secretion of trophic factors that encourage host progenitor cells to undergo tissue regeneration^44^. Indeed, MSCs secrete factors that are both angiogenic, e.g. vascular endothelial growth factors, fibroblast growth factors and fibronectin^10–14^, and also that inhibit endothelial cells and blood vessels formation, e.g. aggrecan^42,43^; therefore further study is required to examine the relative levels of these secreted factors in conditioned medium from PA MSCs in comparison to CD271+ MSCs.

In conclusion, our data shows that PA MSCs and CD271+ MSCs demonstrate similar MSC-like properties, in terms of differentiation capacity, which supports their use in cell-based therapies. However, selective isolation of CD271+ MSCs from adipose tissue results in the culture expansion of a subset of cells that are less angiogenic and were better able to promote cartilage repair of osteochondral tissue damage. Therefore, the transplantation of CD271+ MSCs may be more favoured for the restoration of mature, avascular tissues such as articular cartilage.

## Methods

All methods in human and animal studies were performed in accordance with the relevant institutional guidelines and regulations.

### Isolation and culture of plastic adherent MSCs and CD271 immunopositive MSCs from adipose tissue

Following ethical approval from the National Health Service (NHS) Health Research Authority (LREC number 12/EE/0136) and informed consent from all donors, PA MSCs and CD271+ MSCs were isolated from AT that had been harvested as surgical waste products. The AT was minced and treated with 0.3 U/ml of collagenase (Sigma, Dorset UK) for two hours at 37 °C after which Dulbecco’s Modified Eagle Medium (DMEM) supplemented with 20% (v/v) fetal calf serum (FCS) and 1% (v/v) penicillin and streptomycin (All from PAA, Yeovil, Somerset, UK) was added and the digested preparation centrifuged at 600 g for 10 minutes. The resulting pellet was re-suspended in DMEM supplemented with 10% (v/v) FCS and 1% (v/v) penicillin and streptomycin, i.e., standard culture medium and passed through a 100 μm cell strainer (BD Biosciences, Berkshire, UK). The filtrate was re-centrifuged at 600 g for 10 minutes and pelleted cells re-suspended in 5 ml standard culture medium and passed through a 40 μm cell strainer. The resulting cell suspension was then treated with Erythrocyte Lysis Buffer (Miltenyi Biotech, Surrey, UK) for 10 minutes at room temperature. For each AT preparation, MSCs were isolated from the mononucleated cells present after erythrocyte lysis through their increased adhesion to tissue culture plastic^8^ or by magnetic associated cell sorting (MACS) for CD271 immunopositivity^14^. These cells were culture expanded in standard culture medium in a humidified atmosphere at 37 °C with routine passaging by trypsinisation at 80% confluence. PA MSCs and CD271+ MSCs at passages II-III were used for all subsequent experimentation. Flow cytometry was used to assess the enrichment for CD271+ cells (using MACS technology), where the freshly isolated cells were stored at −80 °C overnight after isolation, and then thawed and immediately immunostained for CD271; for all samples, >90% of cells were CD271 immunopositive at this time point (data not shown).

### *In vitro* differentiation protocols

The differentiation capacity of PA and CD271+ MSCs to form osteocytes, chondrocytes and adipocytes was assessed as described previously^8,45^. Briefly, for osteogenesis, monolayer cultures of MSCs were treated with 10 nM dexamethasone, 50 ng/ml ascorbic acid and 1 mM beta glycerophosphate (versus carrier controls) every 2–3 days for a period of 4 weeks, after which the cultures were harvested by fixation and stained for alkaline phosphatase activity as follows: cells were fixed with 10% neutral buffer formalin for 10 minutes. Meanwhile, staining solution was prepared by placing 25 mg of naphthol-phosphate (Naphthol AS-MX phosphate: Sigma) in 0.5 ml of dimethyl formamide. This solution was mixed with 50 ml of 0.2 M Tris-HCl buffer containing 50 mg of fast red TR (Sigma). After mixing well, the final solution was filtered using Whatman No. 1 filter paper (Whatman). The fixative solution was then removed and cells were washed with PBS, then 1 ml of the staining solution was added to each well for 1 hr. Finally, the stain was removed and digitized images were captured with an inverted microscope. Differentiation along the osteogenic lineage was further evaluated by increased amount of alkaline phosphatase activity in differentiated cells compared to undifferentiated cells using a commercially available kit (Biovision, USA) and by following the manufacturer’s protocol; in brief, cells were homogenized in the assay buffer. The homogenized cells were then centrifuged to remove insoluble material at 13,000 g for 3 minutes. Then 80 µl of each of the samples was loaded into separate wells of a 96 well plate. Then 50 µl of 5 mM of *p*NPP solution was added to each sample well. Following a mixing step by pipetting up and down, the wells were incubated at 25 °C for 60 minutes. A standard curve was generated by diluting 40 µl of 5 mM of pNPP into 160 µl of assay buffer to generate 1 mM *p*NPP standard. Then 0, 4, 6, 12, 16 and 20 µl of this standard solution was loaded into a 96 well plate to generate 0–20 nmol/ml *p*NPP standards that could be measured by spectrophotometry. For chondrogenesis, cell pellets were prepared in DMEM/high glucose (Sigma) supplemented with 100 nM dexamethasone (DEX), 37.5 μg/ml ascorbate-2-phosphate, 1% (vol/vol) insulin, transferrin and selenium (ITS-X100; Sigma) and 10 ng/ml transforming growth factor-β1 (TGF-β1) (PeproTech, London, UK), and penicillin and streptomycin. Control cultures were treated with DMEM/High glucose media with carriers alone, i.e. methanol, sterile water and BSA at appropriate dilutions. At day 28, chondrogenic differentiation was examined histologically by fixing the pellets in 10% neutral buffered formalin, then embedding in paraffin and cutting tissue sections, which were stained with toluidine blue (Sigma) as a marker of proteoglycan synthesis^8^. In addition, the level of glycosaminoglycan secreted into the differentiating medium in the last 24 hours of culture prior to harvesting at day 28 was measured using the DMMB assay. The DMMB assay protocol was adapted from the method of Farndale *et al*., 1986 as follows^46^: (i) the DMMB dye solution was prepared by adding 3.04 g of glycine, 2.37 g of NaCl and 16 mg of 1,9 DMMB to 1 litre of deionized water; (ii) the pH was adjusted to 3.0 with hydrochloric acid and the dye solution was stored in a brown bottle; (iii) 50 μl aliquots of culture medium harvested from the cell-seeded scaffolds at day 28 were added in triplicate to a 96 well plate; (iv) 200 μl of the DMMB dye solution was added to the culture medium and the absorbance was assessed at 540 nm immediately. Chondroitin sulphate (CS) from shark cartilage (Sigma) was used to provide a standard curve of absorbance (0–40 μg/ml, CS) from which the GAG content in the samples of culture medium was calculated. The levels of absorbance for GAG content in the samples of culture medium were normalized to account for the background absorbance resultant from the presence of phenol red within the medium by dissolving the standards in DMEM medium. For adipogenesis, MSC cultures were maintained in culture media containing 1 μM DEX, 0.5 mM 3-isobutyl-methylxanthine (Sigma), 1% insulin, transferrin and selenium (ITS-X 100 pre-mix; PAA) and 100 μM indomethacin (Sigma), for 28 days at 37 °C and 5% CO_2_, as previously described^47^. Control cells were maintained in complete media with carriers. Differentiation was examined using Oil Red O staining of lipid droplets. Cells were fixed in 10% neutral buffer formalin for 1 hour after which the staining solution was added. Relative Oil Red O accumulation was measured after treatment with 100% isopropanol for 15 minutes and reading the absorbance of the supernatant at 540 nm using a spectrophotometer.

### MSC transplantation into a femoral osteochondral defect model

**Figure 6:**
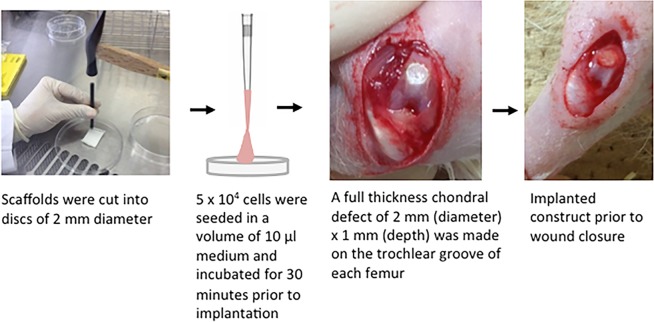
The procedure of MSC transplantation into full-thickness osteochondral defects. Scaffolds were cut into 2 mm diameter sized disks and were sterilised using UV prior to cell-seeding. Culture-expanded PA MSCs and CD271-selected MSCs were trypsinised and seeded onto the Alpha Chondro Shield scaffold using a simple pipetting technique in a volume of 10 µl. Subsequently, the cell-seeded scaffolds were transplanted into the defects and fixed in place using fibrin glue.

Following institutional ethical review and approval (The Institutional Animal Care and Use Committees of Fukui University, Department of Orthopaedics and Rehabilitation Medicine: Approval Number 25–053), female athymic nude rats (F344/N Jcl rnu/rnu, CLEA Japan, Inc. Tokyo, Japan) aged 6–10 weeks and weighing 150–170 grams were randomly allocated into the following groups: (i) PA MSCs (n = 6 animals); (ii) CD271+ MSCs (n = 6 animals); (iii) a control group of Alpha Chondro Shield scaffold alone (no cells, n = 3 animals). These numbers of animals per group have been used previously at the same institute to demonstrate significant differences between treatment groups at the 5% level^48^. The rats were anaesthetized by exposure to 3% isoflurane in O_2_ gas and maintained at 1.5% isoflurane in O2 gas during surgery. After sterilising the knees using 70% ethanol, a medial parapatellar skin incision was made followed by dissecting through the muscle and then exposing the knee joint by lateral dislocation of the patella. Bilateral osteochondral defects of 2 mm diameter and 1 mm depth were created in the patellar groove of the femur of each animal using a 2 mm diameter surgical drill. Alpha Chondro Shield was used as the cell carrier/scaffold to deliver 5 × 10^4^ PA MSCs or CD271+ MSCs versus no cells (as controls). Prior to transplantation, cells were harvested by trypsinisation and seeded in a volume of 10 μl of standard culture medium onto 2 mm diameter disks of Alpha Chondro Shield, then incubated in a humidified atmosphere at 37 °C and 5% CO_2_ for 30 minutes to promote cell adherence and incorporation into the scaffold. The cell-seeded and control scaffolds were transplanted into the defects and fixed in place with fibrin glue, which was allowed to set for about 10–20 seconds (Fig. 6). The patella was relocated, and the connective tissue and skin sutured with nylon sutures. Animals were allowed to move freely following recovery and fed a standard maintenance diet and water *ad libinum*. At 3 weeks post-transplantation, animals were sacrificed by overdose of 3% isoflurane to examine the extent of wound repair macroscopically and histologically.

### Macroscopic scoring of cartilage wound healing

After exposing the knee joint in sacrificed animals, the defect was scored macroscopically by two surgeons who were blinded to the treatment arm using an established system for assessment of cartilage wound healing in smaller animal models^49^. The degree of tissue repair was scored from 0 points (best outcome) to 4 points (worst outcome) each for: (1) the colour of the repair tissue; (2) the extent of blood vessels seen within the repair tissue; (3) the smoothness of the surface of the repair tissue; (4) the extent to which the wound defect was filled with repair tissue; (5) the extent of degeneration of the adjacent articular cartilage. The scored points for each criterion were added and the degree of best repair was represented with the lowest score from a total score of 20.

### Histological scoring of cartilage wound healing

Following surgical excision, animal knees were fixed in 10% neutral buffered formalin (Sigma) for 48 hours and placed in K-CX decalcifying solution (FALMA, Tokyo, Japan) for 24–48 hours at 4 °C. Following this, the rat knees were washed overnight in running tap water, processed and embedded in paraffin wax blocks ready for sectioning. Tissue sections were cut at 5 microns thickness in a standard rotary microtome and these were stained with haematoxylin and eosin (H&E) and toluidine blue prior to mounting in DPX and histological examination. The extent and quality of cartilage repair was assessed histologically and independently by two examiners using the Wakitani scoring system^36^, modified to include two additional parameters, i.e. to examine the presence of blood vessels and foreign body giant cells (FBGCs). Hence, the scoring system comprised of seven different parameters: (1) cell morphology; (2) matrix staining; (3) surface regularity; (4) thickness of cartilage; (5) integration of the repair tissue with host cartilage; (6) the extent of vascularisation within the defect; (7) the extent of FBGC reaction. For each of these parameters, the lowest score (0) represented the ‘best’ repair and a highest score (4) represented the ‘worst’ repair. The overall scores were added and then compared between groups out of a total score of 21.

### Immunohistochemistry for human mitochondrial antigen

Tissue sections were stained with an anti-human mitochondrial antigen (HMA) antibody (Clone 113-1: Abcam, Cambridge, UK) to assess the presence of human MSCs. Following antigen retrieval, the sections were immersed in three changes of PBS and blocked for 20 minutes with 2.5% horse serum (Vector Labs Ltd) at room temperature to prevent non-specific binding. The sections were then incubated with the mouse anti-HMA antibody (1:400 dilution in PBS) for 1 hour at room temperature in a humidified chamber. Following this, any unbound antibody was washed off in three changes of PBS gently and the sections were incubated with biotinylated anti-mouse IgG for 30 minutes at room temperature. The sections were washed three times in PBS and endogenous peroxidise activity was blocked using 0.3% hydrogen peroxide in methanol for 30 minutes at room temperature. During this incubation step, the Vecta ABC regent (Vector Labs Ltd, Peterborough, UK) was prepared and was allowed to stand for 30 minutes before use as per the manufacturer’s instructions. After blocking the endogenous peroxidise activity, sections were washed in three times PBS and incubated with the ABC reagent for 30 minutes at room temperature. Following this, a DAB chromogen (Vector labs) was added for 6–8 minutes depending on the intensity of colour desired. Sections were then washed and dehydrated through series of ethanol (70–100%), cleared with xylene and mounted in Pertex. Chondrogenic pellet of human MSCs was used as a positive control. Negative control included chondrogenic pellet where the primary antibody was omitted.

### Scanning electron microscopy

The adhesion of MSCs seeded into the Alpha Chondro Shield scaffold prior to implantation was examined by scanning electron microscopy (SEM) as follows: after the cell-seeded scaffolds had been incubated in a humidified atmosphere at 37 °C and 5% CO_2_ for 30 minutes, they were washed in PBS and then fixed in 2% glutaraldehyde in 0.1 M phosphate buffer (pH 7.4) for 2 hours, then washed in 0.1 M phosphate buffer prior to treatment with 1% osmium tetraoxide for 1 hour. The samples were then dehydrated through a graded series of ethanol solution, treated with transition solvent, t-butyl alcohol for 30 minutes, freeze-dried, coated with gold palladium and finally imaged using a JSM-6390 (JEOL, Tokyo, Japan) or Zeiss EVO10 scanning electron microscope (Carl Zeiss, Cambridge, UK).

### LIVE/DEAD staining to assay cell viability in cell-seeded scaffolds

MSC-seeded scaffolds were stained with LIVE/DEAD staining solution following the manufacturer’s protocol, as described previously^8^. Briefly, the cell-seeded scaffolds were immersed in the LIVE/DEAD staining solution for 30 minutes in the dark at 37 °C. The scaffolds were then removed from the staining solution, washed in PBS and immediately imaged using a confocal microscope (Leica Microsystems DM6000B – SP57CS).

### *In vitro* angiogenesis assays

The human EA.hy926 endothelial cell line was used as a model to examine any angiogenic activity of culture conditioned medium from PA MSC and CD271+ MSC CM. EA.hy926 endothelial cell proliferation/migration and endothelial tubule formation were examined as follows: (i) EA.hy926 cells were cultured in standard culture medium (DMEM/ F-12 supplemented with 10%FBS, 1% penicillin and streptomycin) in 24 well plates for 2 days until 100% confluent monolayers formed. A sterile pipette tip was used to make a scratch in the monolayer. Subsequently, the wells were washed with sterile PBS and then 1 ml of conditioned media from PA MSC cultures or CD271+ MSC cultures was added into each of triplicate wells per condition tested. Serum-free DMEM culture medium was used as a control. The plate was incubated in the Cell IQ platform for live cell digitized imaging over a 2 day period, after which scratch wound closure, viable cell numbers and the extent of migration of individual cells were quantitated using the Cell IQ image analysis software. The proliferative response of EA.hy926 endothelial cells to PA MSC And CD271+ MSC conditioned media (versus serum-free control medium) was also examined using the MTT assay; (ii) EA.hy926 endothelial tubule formation was tested using growth factor-reduced Matrigel (BD Bioscience), which was aliquoted into 96-well plates (50 μl Matrigel/well) and then incubated for 30 min at 37 °C. EA.hy926 endothelial cells were seeded onto the Matrigel at 2 × 10^4^ cells/well in 200 µl of PA MSC and CD271+ MSC conditioned media or serum-free control culture medium. The plates were incubated at 37 °C for 1 day, after which they were imaged using Cell IQ imaging platform (3 images per well). The total endothelial tubule length/image and total number of endothelial tubule branching points/image were measured using the Cell IQ image analyser software.

### Statistical analysis

Statistical analysis was performed using GraphPad Prism6 software (GraphPad Software, Inc. CA, USA). For *in vivo* experiments, a minimum of n = 3 animals per condition were tested. For *in vitro* experiments, n = 3–4 independent experiments were performed for all analyses, where data were analysed with one-way ANOVA when there was one variable factor, and two-way ANOVA when there were two variable factors. For macroscopic and histological scores of cartilage repair, differences between groups were examined using Dunn’s multiple comparison tests. A *p* value of less than 0.05 was considered significant. All data has been presented as means ± SEMs or means ± SDs (as indicated in the figure legends).

## Data Availability

Data generated and analysed during this study are included in this published article. Data and materials are available from the corresponding author subject to reasonable request and subject to the ethical approvals in place and material transfer agreements.
